# Actinomycosis as a Rare Local Manifestation of Severe Periodontitis

**DOI:** 10.1155/2020/5961452

**Published:** 2020-02-01

**Authors:** Moody Kaldas, André Barghorn, Patrick R. Schmidlin

**Affiliations:** ^1^Private Practice, Oetwil am See, Switzerland; ^2^Clinic of Conservative and Preventive Dentistry, Center of Dental Medicine, University of Zurich, Switzerland; ^3^Institute of Pathology Medica, Zurich, Switzerland

## Abstract

Actinomycosis is a chronic suppurative infection primarily caused by anaerobic gram-positive filamentous bacteria, primarily of the genus Actinomyces. Oral-cervicofacial actinomycosis is the localization found most often, presenting as a soft tissue swelling, an abscess, a mass lesion, or sometimes an ulcerative lesion. Periodontitis-like lesions, however, are rare findings. This report describes the case of a 41-year-old healthy female patient (nonsmoker), who was referred to the clinic with dull and throbbing pain in the second quadrant. Tooth 25 showed increased mobility and probing pocket depths up to 10 mm, with profuse bleeding upon probing. Radiographically, considerable interproximal horizontal bone loss was found, and the diagnosis of periodontitis stage 3, grade C was made. The situation was initially stabilized with adhesive splinting and local anti-infective therapy. Two weeks later, the bone defect was treated with guided tissue regeneration (GTR) using a xenogenic filler material (BioOss Collagen) and a resorbable membrane (Bio-Gide). Due to a suspicious appearance of the excised granulation tissue, the collected fragments were sent for histopathological evaluation. This evaluation revealed a chronic granulomatous inflammation with the presence of filamentous bacterial colonies, consistent with Actinomyces. The patient was successfully treated. While there are only few reports in the literature, actinomycotic lesions represent a rare but possible finding in cases with localized periodontal destruction. In conclusion, systematic biopsy of the infrabony tissue in localized periodontal lesions may help to provide a more accurate counting of Actinomyces-associated lesions, thereby improving diagnosis, therapy, and prevention.

## 1. Introduction

Actinomycosis is a chronic infectious disease, usually characterized by abscess formation and draining sinuses. It is known for more than a century to be caused mainly by gram-positive filamentous bacteria, which have a fungus-like histomorphological appearance. Typical intertwining bacterial filamentous colonies can be found, which are called sulfur granules because they macroscopically appear as yellowish specks [1]. Since these bacteria are frequently found in the mouth, nose, and throat regions, it is not surprising that actinomycosis mostly affects the face and neck regions. Cervicofacial actinomycosis accounts for about 50-60% of the cases [2]. Parotid and submandibular glands and the mandible are mostly affected [3]. The so-called “lumpy jaw” includes both soft and hard tissue of the head and neck regions [4]. Other localizations include the chest (pulmonary actinomycosis), abdomen, and pelvis. The bacterial infection always involves several genera of bacteria, which synergistically interact. However, the disease may not be diagnosed correctly unless the tissue is biopsied and the Actinomyces colonies are identified [4]. Actinomycosis is four times more common in men than in women and affects especially men between 20 and 60 years of age [5].

For disease establishment, bacteria must be able to enter and colonize tissues; for instance, due to trauma, surgery, or other common infection pathways. If an abscess occurs, the infection drains mostly through the skin and generates a sinus tract. Common accompanying symptoms are fever, mild or no pain, swelling or a hard, red to reddish-purple lump on the affected site. It is noteworthy that different Actinomyces strains exist in the physiologic and pathogenic oral flora [6]. Studies have shown coaggregations between strains of *A. viscosus* and *A. naeslundii* with oral streptococci-like *S. mitis* [7–9]. Specific morphological arrangements in the form of corncobs have also been observed [10, 11], which are mainly mediated by cell-surface interactions between bacterial receptors [8, 9]. Under physiological symbiotic conditions, these bacteria are commensals and do not cause pathologies. However, given specific dysbiotic circumstances, tissues may be damaged, invaded and opportunistic chronic or aggressive inflammatory processes may be initiated. In general, the functional integrity of the mucosal barrier or the periodontal ligament must be somehow compromised, allowing for access to the underlined tissues [12, 13]. Among the isolated Actinomyces species, *A. israelii* is the most common identified strain in actinomycosis. Coaggregated cells are more resistant to phagocytosis and killing by neutrophils and are therefore linked with pathogenicity [14].

In this report, we present a rare case of localized periodontal destruction between teeth 25 and 26, which was ultimately diagnosed as an actinomycosis (or Actinomyces-associated lesion) based on histopathology, although the clinical presentation resembled a simple localized severe periodontitis. It is noteworthy that approximately 14 percent of rare diseases with orofacial involvement also show manifestations in the form of gingivitis, periodontitis, and gingival hyperplasia in connection with the underlying disease [15]. However, the new classification of periodontal and peri-implant diseases and conditions does not consistently mention or classify them. Also, actinomycosis was not included and discussed. Therefore, we aim to highlight and describe the characteristics of this particular lesion, discussing the relevant literature and potential implications for future research.

## 2. Materials and Methods

### 2.1. Case Report

A 41-year-old female patient was referred by her private dentist to our clinic for a second opinion and treatment of a localized interdental periodontal bone defect between teeth 25 and 26. The patient was systemically healthy and a nonsmoker. Over the course of the previous two years, she had regularly attended maintenance appointments with her dentist and dental hygienist. A little over one year ago, a loss of bone level was observed; however, local therapy (scaling and root planning under local anesthesia) neither brought stagnation nor improvement. At this point, the periodontal referral was initiated.

At the time of the first evaluation, the patient was not suffering from acute pain. However, the patient reported an intermittent throbbing sensation in the interdental space and a feeling of pressure.

**Figure 1:**
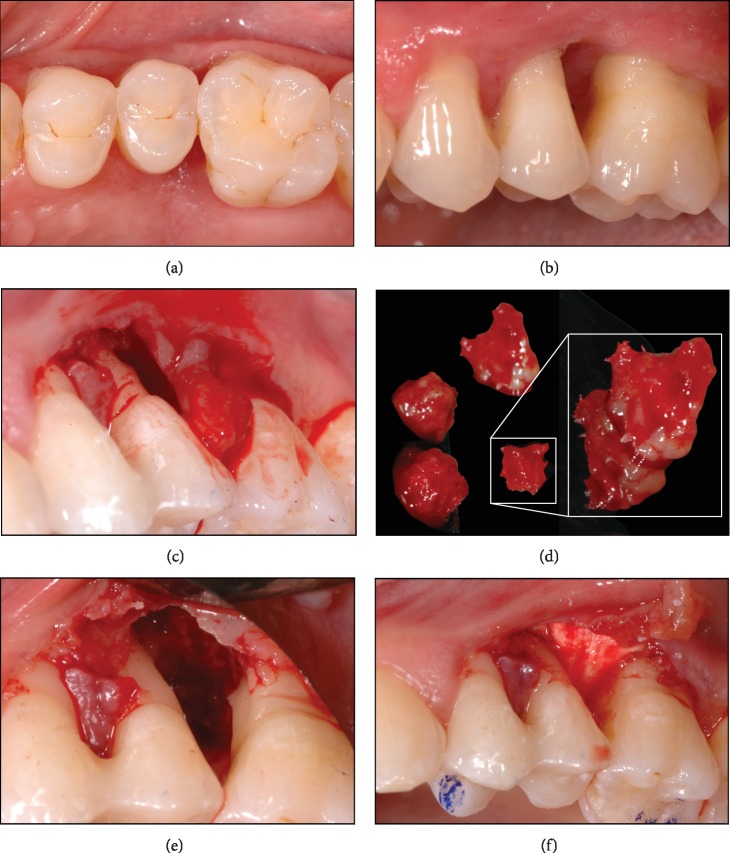
Clinical images of the case before therapy (a, b). A periodontal flap was raised (c), and granulation tissue was removed (d). The defect (e) was filled with a xenogenic material and covered with a resorbable membrane (f).

The patient displayed overall excellent oral hygiene (very minute visible plaque and calculus, if any), but—as a secondary sign of accentuated brushing habits—some recession, on the buccal surfaces, as well as initial noncarious cervical lesions (mixed erosions/abrasions). Intermittently, the patient noted some gingival bleeding, especially when using dental floss at this site. Tooth 25 showed increased tooth mobility (Grade 3) and probing pocket depths up to 10 mm, with profusive bleeding upon probing. Neither tooth 25 nor 26 had probable furcations. The periodontal screening indices (PSI) of the other sextants displayed values of a maximum of 2, which corresponded to no probing pocket depths greater than 3 mm and maybe some bleeding and even minute calculus (Figures 1(a) and 1(b)). In this context, some interdental attachment loss of the other teeth was up to 4 mm (>5 mm in the affected area).

**Figure 2:**
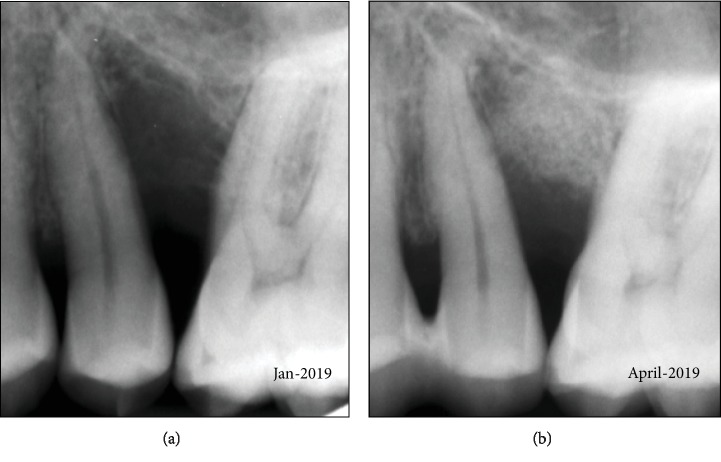
Radiographs before (a) and after surgery (b).

The percussion test for teeth 25 and 26 was negative, and their vitality test positive (CO_2_). Clinically, a missing papilla was noted. The col area was keratinized, and no signs of ulceration or necrosis were visible. The loss of tissue was radiographically corroborated, where a clear bone deficiency was visible (Figure 2(a)).

#### 2.1.1. Diagnosis, Prognosis, and Treatment Plan

A localized severe periodontitis stage 3, grade C was diagnosed (formerly known as “localized aggressive periodontitis”). Since the patient was highly motivated, displayed good oral hygiene, had no furcation involvements, and was in good systemic health, with no history of smoking, the overall prognosis for therapy was considered to be good. The fact that an experienced dental hygienist had already performed subgingival scaling without any clinical success and subgingival calculus was not identifiable, we put a surgical intervention and focus to open inspection. A root fracture could also not be excluded, and therefore, explorative flap surgery was discussed with the patient. She was informed about all therapeutic options, i.e., primary, an open flap debridement, potentially with a regenerative procedure, materials, and costs. The regeneration of the defect was mainly dependent on the bony characteristics of the lesion (defect architecture, depth, and containing walls). A treatment plan consisting of xenogenic filler material application in combination with enamel matrix proteins and possibly fixing a membrane, if applicable, was determined. Intra- and postoperative risks were highlighted (mainly, infection and incomplete regeneration). The patient agreed to a surgical intervention with a regenerative approach (details of the respective intervention as actually performed will follow below). No bacterial test was performed due to the local characteristics of the lesion, and since a prescription of antibiotics has inherent risk factors, as such, the test was not planned at that stage.

#### 2.1.2. Treatment

Due to the tooth mobility and functional overload of the involved teeth, an interdental splinting with composite resin material including occlusal adjustment was indicated and performed 2 weeks before surgery. Tooth 25 was even left in slight infraocclusion. Preoperatively, the patient received 500 mg mefenamic acid (Ponstan, Pfizer, Zurich, Switzerland). Under local anesthesia, a modified papilla preservation flap at the interdental papilla on the palatal site was prepared (surgery: PRS; Figure 1(c)). In order to avoid vertical incisions, the flap was extended to the mesial and distal aspects with a semilunar incision at the buccal site. A mucoperiosteal flap was prepared, and the defect thoroughly debrided of granulation tissue. The latter was found to be of a very unique consistency as compared to excised/removed periodontal granulation tissue, which normally consists merely of soft tissue. However, the removed material contained filamentous and relatively hard granular particles (Figure 1(d)). Therefore, a histological analysis of the solid collectable tissues was deemed advisable and this tissue was transferred to a biopsy vial. Afterwards, the tooth surfaces were thoroughly cleaned and the defects abundantly rinsed with PVP iodine (Betadine, Mundipharma) [16]. Afterwards, the defect was inspected for tissue and calculus remnants (Figure 1(e)). A fracture of the root or other hard tissue-related complications such as cemental tears were excluded [17]. The area was rinsed with sterile saline solution and dried with swabs. Then, enamel matrix derivatives (EMD, Straumann, Basel, Switzerland) were added to the dry tooth surface and a mixture of 90% bovine bone granules with the addition of 10% porcine collagen (Bio-Oss Collagen, Geistlich, Wolhusen, Switzerland) was filled into the defect and adapted. The latter had been soaked with EMD before application for 5 minutes. Finally, a resorbable collagen membrane (Bio-Gide, Geistlich) was applied to cover the filled defect (Figures 1(f) and 2(b)) and the defect closed. For prophylactic measures, clindamycin (Dalacin C, Pfizer, Zurich, Switzerland) was prescribed for 7 days with a suggested intake 3 times 300 mg per day. Pain killers (mefenamic acid, Ponstan) were prescribed for the first two days, if needed.

The biopsy samples were fixed in 4% neutral-buffered formalin solution and processed according to standard protocols. The slides were stained with hematoxylin and eosin (H&E), elastica van Gieson (EvG), periodic acid Schiff's reaction (PAS), Gram, and Giemsa using a Tissue-Tek Prisma stainer module (Sakura Seiki, Nagano, Japan) and commercial staining solutions. Additional stains included Grocott and Ziehl-Neelsen executed on a VENTANA BenchMark Special Stains stainer module (Roche Diagnostics, Risch-Rotkreuz, Switzerland) using the manufacturers' GMS II Staining Kit and AFB III Staining Kit, respectively.

After one week of uneventful healing, sutures were removed, proper oral hygiene was reinforced, and the patient was instructed to continue rinsing with antiseptic up to 6 weeks.

## 3. Results

### 3.1. Histologic Evaluation

**Figure 3:**
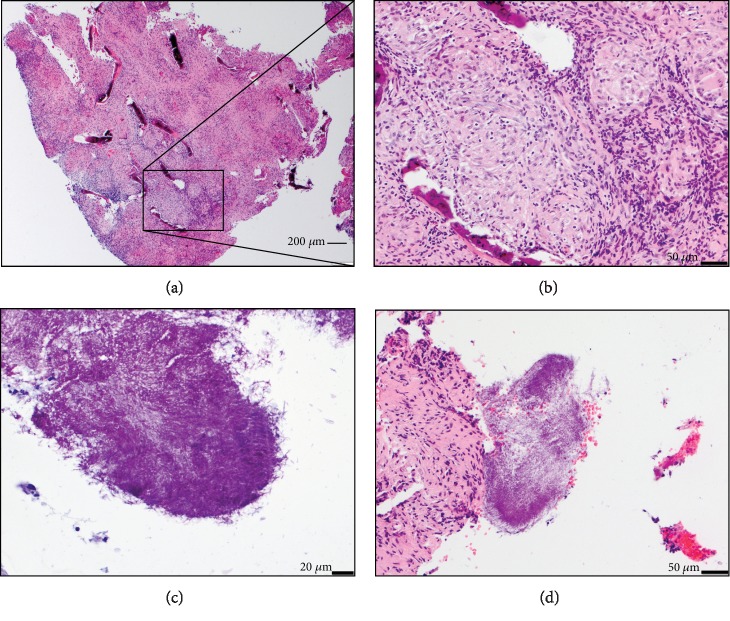
Histology of a representative tissue fragment with clearly visible bone trabeculae and neutrophil infiltration (a), at higher magnification (b). Also visible were different colonies of bacteria (mainly Actinomyces spp.) between and adherent between the tissue fragments (c, d).

The biopsy samples showed an extensive epithelioid granulomatous reaction with Langhans giant cells, fibrosis and 4n destruction and focal remodeling (Figures 3(a) and 3(b)). Fragments with squamous epithelium demonstrated intraepithelial neutrophilic infiltration and subepithelial aggregations of lymphocytes and abundant plasma cells. On the surface of the biopsy, colonies of filamentous bacteria were identified (Figures 3(c) and 3(d)). These were positive for gram and PAS stains but not for Ziehl-Neelsen' stain, consistent with Actinomyces spp. Acid fast bacteria were not detected.

### 3.2. Clinical Outcome

**Figure 4:**
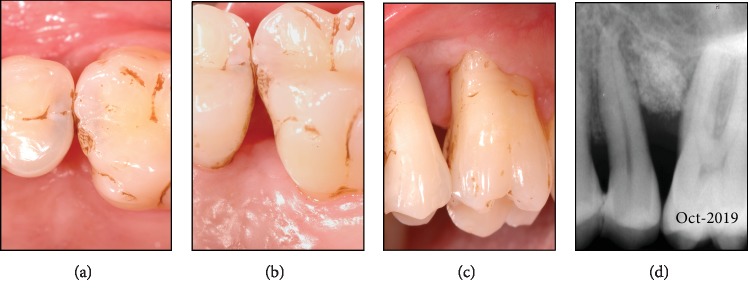
Clinical outcome revealed no residual pockets > 3 mm and no bleeding on probing; some staining was visible due to rinsing with chlorhexidine (a–c). The radiograph showed a densification of the grafted site and stable conditions (d).

No probing was performed until six months postoperative. At this reevaluation point, no probing depths exceeding 3 mm were found and no bleeding upon probing was observed. The interdental soft tissues displayed adequate keratinization, with no signs of inflammation (Figures 4(a)–4(c)). The radiograph showed a consolidation of the implanted material, with some accentuated mineralization (Figure 4(d)).

## 4. Discussion

The case presented showed clinical features which were diagnosed as a severe periodontitis stage 3, grade C. Extensive localized attachment and bone loss were apparent and were corroborated by radiographs. A histologic evaluation of the excised granulation tissue ultimately revealed the presence of gram-positive filamentous bacterial colonies, consistent with Actinomyces spp. The question as to whether local trauma and/or infection caused disease initiation and progression between the two teeth in the absence of actual systemic or environmental risk factors remains speculative. Lesions related to actinomycosis have been described previously in a few periodontitis case reports (Table 1) [2; 18-20].

Despite previous data showing that actinomycosis is more common in men than in women [5], all identified publications reported on cases in females, with one case in a very young patient, but all clinically diagnosed with periodontitis. Unfortunately, the new classification of periodontal and peri-implant diseases and conditions does not mention it. It seems, therefore, important to have actinomycosis as a potential distinct pathway of localized periodontitis in mind, which could be—at least—considered an atypical presentation of local tissue destruction resembling periodontitis. It may even be a distinct disease entity. Oral actinomycosis is not a common disease, but it can cause massive destruction. Interestingly, implant failure mimicking peri-implantitis associated with actinomycosis has also been reported in the literature as well [21].

As always—when looking at isolated destructive processes—the question arises as to which specific (local) causes can explain such devastating tissue involvement. Several explanations for isolated defects have been described in the literature: vertical root fractures [22], endoperiolesions [23], functional overload [24], foreign body reactions [25], and cemental tears [16, 26]. Clinicians must always be aware of such potential implications, but a clear identification of the causative agent is sometimes very difficult clinically. Periodontitis in younger patients, formerly known as early-onset, juvenile, or aggressive periodontitis also reveals distinct patterns of local or generalized, but sometimes symmetric involvement of teeth and sites. The reason for this remains unclear; however, age at onset, the duration, and the frequency of systemic factors give rise to a possible distinction between localized or more generalized forms [27]. Obviously, bacterial colonization of the tissues remains the primary or major cause for attachment loss. The composition and grade of infection may be influenced by systemic, environmental and local factors as mentioned above [28]. Especially when facing local infestations, the role of a true infective process becomes more prominent [29]. Actinomycosis, a chronic infection primarily caused by gram-positive anaerobic bacteria, can display acute and chronic infective features.

The organisms may histologically display a number of fungus-like characteristics, i.e., a tendency to grow as mass of rounded bodies (clubs) and filaments. Due to a rather low intrinsic virulence, a chronic granulomatous tissue response is frequent. Acute forms normally display a rather sudden onset and one or more multiple pus discharging sinuses containing sulphur granules. In the case of periodontitis-like lesions, the sulcus and pocket may be considered a draining surrogate and the progression and clinical presentation may be completely different.

Our case should be considered a gradual onset with typical signs of inflammation, which led to bone involvement and loss with fibrosis rather than suppuration, which renders a proper clinical diagnosis even more difficult. Actinomycosis is considered one of the most “misdiagnosed diseases” and is listed as a “rare disease “by the National Institute of Health (NIH) [30]. Its implication in periodontitis has been vastly neglected so far.

However, reports of cases are growing—given a global look at actinomycosis according to a systematic review of pediatric cases—which showed 10 reports were made during the past 11 years, while only 20 cases were described in a 48-year period [31]. This was mainly attributed to more modern diagnostic techniques.

When it comes to treatment of Actinomyces-associated lesions, removal of the foci of infection including the excision of all involved granular soft tissue and sequestrated bone is mandatory. Penicillin administration has also recommended and showed better in vitro activity as compared to other antibiotics including clindamycin [32]. In the case of periodontitis, the sole prescription of penicillin is not a frequent therapeutic scheme; however, it is frequently prescribed in orofacial infections [33, 34]. As this case showed, removal of the affected tissues and proper debridement resulted in an adequate healing response. In the present case, clindamycin was mainly prescribed as a bacteriostatic agent in order to avoid (re) infection of the implanted materials. Since chronic infections may include aerobes and anaerobes, clindamycin may have a role in the therapy of these infections; however, the spectrum against the main putative periodontopathogens remains rather low, but it has been described as an alternative for treatment of several head and neck infections.

One important question remains, namely, how the diagnosis of “actinomycosis-” or “Actinomyces-associated lesion” can actually influence the treatment, since it can only be provided after tissue removal during surgery and after histological evaluation, which may take some time. This implies that at the day of surgery, important information may still be missing. If there is a difference in selective treatment options, i.e., the standard protocol of localized periodontal destruction with or without Actinomycosis infection remains to be elaborated and more epidemiological data on potential implications of this rare disease in periodontology have to be gathered.

## 5. Conclusions

Actinomycosis infection should be considered in case of an uncommon localized destruction in the periodontium, and biopsies including histopathologic evaluations should be envisaged.

## Figures and Tables

**Table 1:** Overview on the available case reports.

Study	Clinical diagnosis	Histology	Age/sex	Tooth/teeth	Systemic diseases	Therapy
[2]	Adult periodontitis, local abscess	Yes	60/f	33/34	n.a.	Debridement, doxycycline, chlorhexidine
[18]	Localized periodontitis	Yes/no images shown	38/f	26/27	Depression	Debridement, excision, amoxicillin
[19]	Chronic periodontitis	Yes	46/f	46	Rheumatic mitral insufficiency	Scalin & root planning, flap surgery with GTR, amoxicillin with clavulanate
[20]	Juvenile periodontitis	Yes	14/f	33/34	Epilepsy	Excision, penicillin
This study	Localized severe periodontitis stage 3 and grade C	Yes	41/f	25/26	Healthy	Flap surgery and GTR

n.a.: not available; f: female; GTR: guided tissue regeneration.
